# Marfan syndrome in childhood: parents’ perspectives of the impact on daily functioning of children, parents and family; a qualitative study

**DOI:** 10.1186/s12887-019-1612-6

**Published:** 2019-07-29

**Authors:** Jessica Warnink-Kavelaars, Anita Beelen, Sarah Dekker, Frans Nollet, Leonie A. Menke, Raoul H. H. Engelbert

**Affiliations:** 1 Amsterdam UMC, University of Amsterdam, Rehabilitation, Amsterdam Movement Sciences, Meibergdreef 9, Amsterdam, Netherlands; 2 Amsterdam UMC, University of Amsterdam, Pediatrics, Meibergdreef 9, Amsterdam, Netherlands; 3 Center of Excellence in Rehabilitation Medicine, Brain Center Rudolf Magnus, University Medical Center Utrecht, and De Hoogstraat Rehabilitation, Utrecht, the Netherlands; 4 Department of Rehabilitation, Physical Therapy Science & Sports, Brain Center Rudolf Magnus, University Medical Center Utrecht, Utrecht, the Netherlands; 5 ACHIEVE, Center of Applied Research, Amsterdam University of Applied Sciences, Faculty of Health, Amsterdam, the Netherlands

**Keywords:** Marfan syndrome, Children, Parents, Family, International Classification of Functioning, Disability and Health for Children and Youth, Participation, Connective tissue diseases, Hypermobility, Pediatrics, Rehabilitation

## Abstract

**Background:**

Marfan syndrome (MFS) is a heritable connective tissue disease caused by a defect in *FBN1*. The diagnosis is based on the revised Ghent criteria. The main features involve the cardiovascular, musculoskeletal, ophthalmic, pulmonary systems and facial features. Although the clinical manifestations of MFS in children are thoroughly addressed in several studies, literature on the impact of MFS on daily functioning is restricted to pediatric advice on sports and leisure participation. Therefore, the full impact of MFS on daily functioning remains unclear. The aim of this qualitative study was to explore parents’ perspectives on the impact of MFS on daily functioning of children with MFS aged 4–12 years, themselves and family regarding functional performance, activities, participation, personal and environmental factors, and disease burden.

**Methods:**

In this qualitative study parents participated in individual semi-structured interviews (*n* = 10) and 3 focus groups (*n* = 5, *n* = 5 and *n* = 6). Meetings were transcribed, and data were analyzed using thematic analysis. Meaningful concepts were coded, and concepts concerning children with MFS were linked to the International Classification of Functioning, Disability and Health for Children and Youth. Thereafter themes were identified and interpreted.

**Results:**

Parents reported their children could not keep up with peers because of fatigue, pain and physical impairments. Children experienced participation restrictions in school, sports, play and other leisure activities. Parents reported their child as being different due to physical appearance, which provoked unsupportive attitudes. Parental burden was caused by high care needs, lack of support, a limited social life, and concerns about the child’s development. Family burden was caused by adjusted and complex family schedules, other family members with MFS, and reproductive planning decision-making, whereas family cohesiveness and caring were positively perceived factors.

**Conclusions:**

Parents perceived a large impact of MFS on daily functioning of their children with MFS, themselves and their family. More awareness among all professionals involved in the care of children with MFS and their families is needed so that professionals can address their support needs and provide tailored interventions, rehabilitation and/or educational programs to empower and improve daily functioning of the children, parents and family.

**Electronic supplementary material:**

The online version of this article (10.1186/s12887-019-1612-6) contains supplementary material, which is available to authorized users.

## Background

Marfan syndrome (MFS) is a heritable connective tissue disease caused by a defect in *FBN1*. The diagnosis is based on the revised Ghent criteria [1]. The main features involve the cardiovascular (aortic aneurysm, mitral valve prolapse), musculoskeletal (increased arm span/height and reduced upper/lower segment ratios, arachnodactyly, hypermobility, scoliosis, hindfoot valgus, pes planus, pectus excavatum and carinatum), ophthalmic (ectopia lentis, severe myopia) and pulmonary (pneumothorax) systems, skin striae and facial features (dolichocephaly, enophthalmos, downward slanting of the eyes, malar hypoplasia and retrognathia) [2–7]. Although the clinical manifestations of MFS in children are thoroughly addressed in several studies, literature on the impact of MFS on daily functioning is restricted to pediatric advice on sports and leisure participation, based on expert opinions and cardiovascular research [3, 5] and a study on pediatric quality of life [8]. Therefore, the full impact of MFS on daily functioning of children, parents and family remains unclear.

This lack of knowledge hampers health care professionals and multi-disciplinary teams who are involved in the care of children with MFS and their families from providing evidence-based advice to parents and children asking about the everyday consequences of MFS and available interventions to improve daily functioning. However, some clues can be drawn from studies on adolescents and adults with MFS, where physical impairments such as pain, fatigue, aortic dissection and skeletal malformations were reported as negative factors for physical activities, psycho-social development, education, work and family life [9–15], as well as from studies on children with other chronic and connective tissue diseases, which reported difficulties regarding daily functioning and quality of life [16–20].

The three objectives of this present qualitative study are to explore parents’ perspectives on the impact of MFS on daily functioning of (1) their children with MFS aged 4–12 years, (2) themselves, and (3) their family. These new insights may provide greater awareness of the broad impact of MFS on daily functioning among all professionals involved in the care of children with MFS and their families, and help health care professionals better address patients’ support needs.

## Methods

### Participants and sampling strategy

All participants were parents of a child with MFS aged 4–12 years. Having one or more children with MFS, in a different age group, was no exclusion criteria. Parents for the individual interviews were recruited from the Expertise Center MFS for Children and Youth of the Amsterdam University Medical Centers, Amsterdam, the Netherlands by an invitation letter. They were selected purposively for diversity of gender and parental diagnoses of MFS. This strategy allowed diverse parental perspectives and experiences regarding the impact of MFS on daily functioning to be captured. Saturation of the data was expected after a sample size of 7–10 interviews. This point in data collection, when no new additional data are found that develop new themes, was based on the literature, the complexity of the research question, the interview topic guide and diversity of the sample [21]. Focus group discussions were announced on the Dutch MFS Patient Association website. Parents of a child with MFS age 4–12 could sign up by sending an email. They were not selected purposively.

### Interviews and focus groups

To collect data on the same topic, we performed individual semi-structured interviews as well as focus groups. This method was used to assure identified themes and to capture different dimensions of the same topic in order to develop a comprehensive understanding of parents’ perspectives on the impact of MFS on daily functioning of their child, themselves and their family [22]. The main framework for a semi-structured conversation for both the interviews and focus groups was a newly developed preset list of open questions based on topics collected from clinical experience and relevant literature (see Table 1) [9–14, 16–19, 23]. A supplementary topic list was provided to obtain more in-depth answers (see Table 1). We checked for the newly developed preset list of open questions and the supplementary topic list with a parent and a psychologist, expert in qualitative research. These checks did not result in alterations. The semi-structured interviews and two focus groups were conducted by a pediatric rehabilitation physician (JW-K, MD, female). One focus group was conducted by a pediatrician (LM, MD, PhD, female). Both professionals had experience with MFS patient care and were trained in interviewing techniques. The interviews lasted between 30 and 70 min and the focus groups lasted 90 min. Fieldnotes were made during and after the interviews and focus groups. Audio recordings were fully transcribed and anonymized.

**Table 1:** Question guide for semi-structured interviews and focus groups

Questions	Semi-structured topic list (ICF-CY)
What do you know about MFS?	
What does your child know about MFS?	
Which features of MFS does your child have? Which features does your child recognize or mention?	mental functions (b1, s1) sensory functions and pain: vision (b2, s2) voice and speech functions (b3, s3) cardiovascular system, immunological and respiratory functions: fatigue (b4,s4) functions of the digestive, metabolic and endocrine system (b5, s5) genitourinary functions (b6, s6) neuromusculoskeletal and movement related body structures and functions (b7, s7) functions of the skin and related structures (b8, s8)
Can you describe the impact of MFS on daily functioning of your child?	learning and applying knowledge (d1) general tasks and demands (d2) communication (d3) mobility (d4) self-care (d5) domestic life (d6) interpersonal interactions and relationships (d7) major life areas: education, school (d8) community, social and civic life: leisure, sport, playing (d9) personal factors: psycho-social development, coping, self-confidence, self-esteem
What kind of physical or emotional support does your child receive?	products and technology (e1) natural environment (e2) support and relationships (e3) services, systems and policies (e5)
What is the attitude of peers and other people towards your child, you and your family?	attitudes (e4)
What are your child’s concerns regarding MFS? How is your child’s coping, self-esteem and self-confidence?	
What current and future concerns do you have regarding your child?	physical impairments (b,s) activity limitations (d) participation restrictions (d) environmental factors (e) personal factors
What is the impact of MFS on your own life?	
What is the impact of MFS on your family life?	
Which care or support do you need for your child, yourself and your family?	

*ICF-CY* International Classification of Functioning, Disability and Health for Children and Youth. The ICF-CY uses an alphanumeric coding system. The letters used are “b” for Body Function, “s” for Body Structures, “d” for Activities/Participation and “e” for Environmental Factors and are followed by a numeric code that starts with the chapter number of one digit

### Data analysis

A thematic analysis approach [24] was used to analyze the data. Transcripts of the individual interviews and thereafter the transcripts of the focus groups were coded independently and consecutively by 2 of the 3 investigators (JW-K, MD, female; SD, MD, female; AB, PhD, female) using qualitative analysis software (MAXQDA 12 sfqda, 1989–2018, VERBI Software – Consult – Sozialforschung GmbH, Berlin, Germany [25]). Codes concerning children with MFS were linked to the International Classification of Functioning, Disability and Health for Children and Youth (ICF-CY) categories [23] according to published linking rules [26]. The ICF-CY offers a conceptual framework and a common language and terminology for recording problems manifested in childhood and adolescence involving body functions and structures, activity limitations, participation restrictions, and environmental factors important for children and youth. With its emphasis on functioning, the ICF-CY can be used across disciplines (clinicians, educators, policy makers, family members and researchers) to define and document the health, functioning and development of children and youth [23, 27]. The intercoder agreements were calculated in qualitative analysis software (MAXQDA 12 sfqda, 1989–2018, VERBI Software – Consult – Sozialforschung GmbH, Berlin, Germany [25]) and served primarily to improve codes and coding instructions. During the process of data collection, themes were identified and validated in successive interviews until data saturation.

### Techniques to enhance the quality of the qualitative data (trustworthiness)

Three investigators (JW-K; SD; AB) ensured the quality of the qualitative research data throughout the data collection and analysis processes [22]. Investigator triangulation, a powerful technique that facilitates credibility (validation) of data through cross verification from two or more sources or researchers was used to ensure rigor [22]. Member checking was performed by asking parents for feedback on the identified and interpreted themes. Expert checking was performed with the members of the Marfan Europe Network during their yearly conference and with members of the board of the Dutch MFS Patient Association [22]. Feedback was added to in the final analysis thereby improving credibility [22]. Standards for Reporting Qualitative Research [28] and Consolidated Criteria for Reporting Qualitative Research (see Additional file 1) [29] assisted during protocol and manuscript preparation.

## Results

### Participant characteristics

Of the 11 parents approached for consent, 10 participated in an individual semi-structured interview conducted between October 2016 and March 2017 (see Table 2). One parent did not participate due to a lack of time. Sixteen parents enrolled in focus group meetings at the yearly MFS patient contact day in April 2017. Two parents participated in an interview as well as a focus group (see Table 2).

**Table 2:** Parent characteristics of individual semi-structured interviews with 10 parents and 3 focus groups with 16 parents (*n* = 5, *n* = 5, *n* = 6)

	Interviews N	Focus groups N
Gender (male/female)	4/6	7/9
Age parents (range) (years)	41 (32–49)	39 (32–55)
Parents with MFS (male/female)	3/2	3/4
Married	9	15
Divorced	1	0
Living together	0	1
Couples (parents of the same child)	2	4
Total children	19	24
Children with MFS	8	16
Age children with MFS (range) (years)	8 (4–12)	8 (2–16)

*N* number; no missing data

### Interviews and focus groups

All individual semi-structured interviews were conducted at the Amsterdam University Medical Centers. Data saturation was reached after 7 interviews, and no additional material or themes were identified in the 3 successive interviews. Thereafter, enrollment stopped. Then, three focus groups were conducted with parents (*n* = 5, *n* = 5 and *n* = 6).

**Fig. 1:**
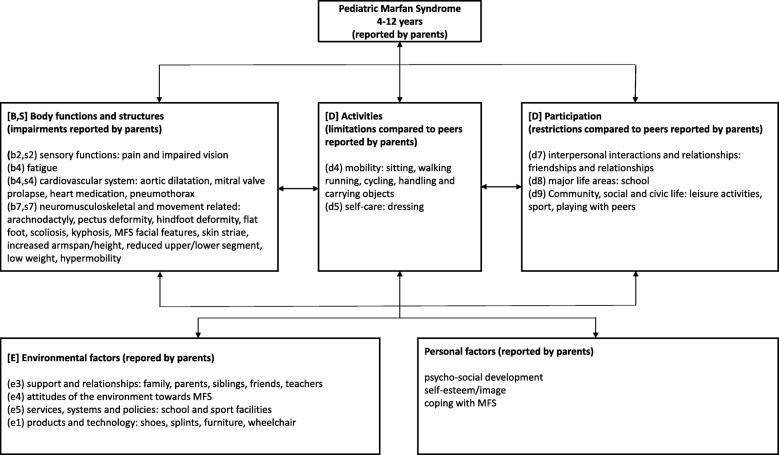
The International Classification of Functioning, Disability and Health for Children and Youth (ICF-CY) diagram: Pediatric Marfan Syndrome 4–12 years; Parents’ perspectives of the impact on daily functioning of children with MFS. Parents’ meaningful concepts concerning their children with MFS were linked to ICF-CY. The ICF-CY uses an alphanumeric coding system. The letters used are “b” for Body Function, “s” for Body Structures, “d” for Activities/Participation and “e” for Environmental Factors and are followed by a numeric code that starts with the chapter number of one digit [23, 26, 27]

The intercoder agreement for the interviews was high for code existence and code frequency (mean [range]: 91.4%, [89.3–94.2%] and 87.9%, [82.3–92.0%], respectively). The intercoder agreement for the focus groups was also high for code existence and code frequency (mean [range]: 81.9%, [80.3–83.3%]; 76.0%, [75.4–76.4%], respectively). The analysis of the semi-structured interviews identified 2 key themes and 10 subthemes on parents’ perspectives on the impact of MFS on daily functioning of children with MFS aged 4–12 years (see Fig. 1 and Additional file 2), 2 key themes and 4 subthemes on parents’ perspectives on the impact of a child with MFS on parental life (see Additional file 2) and 3 key themes and 2 subthemes on parents’ perspectives on the impact of a child with MFS on family life (see Additional file 2). Thereafter, the analysis of the 3 focus groups verified all identified themes and subthemes and no new themes were identified.

Subsequently, both member and expert checks endorsed all themes, and the expert checks added the importance of acknowledgement of the subtheme: “psycho-social development and behavioral problems in children with MFS”. Key themes and subthemes are presented in Additional file 2, where they are supported by quotes from the parents.

### Key themes

#### Parents’ perspectives on the impact of MFS on daily functioning of children with MFS

##### Cannot keep up with peers

Parents reported that their children could not keep up with peers due to fatigue, pain and physical impairments and experienced participation difficulties in school, sports, play and other leisure activities. Limitations in mobility (sitting, walking, running, throwing balls, carrying and preschool fine motor skills), self-care (dressing and eating) and the deterioration of functional performance over time were addressed. These limitations interfered with their child’s participation opportunities. A mother (interview 9) shared,

“He becomes tired much faster than peers; for example, when he goes shopping, he stops everywhere to rest on benches, on poles, in shops. So, he can’t be out for more than an hour.”

##### Being different

Parents perceived their child as being different and standing out from their peers due to their physical appearance related to MFS. Clinical features such as tall stature, low body weight, long and slender limbs, arachnodactyly, hypermobility, scoliosis, pectus excavatum and carinatum, pes planus, genu valgum, facial features and supportive devices (splints and wheelchair) were addressed. Due to tall stature, the children’ ages were often overestimated, leading to comments and reprimands from adults and peers about “childish and clumsy” behavior.

Regarding tall stature, one father (interview 6) stated, “Yes, I’ve been very angry and annoyed by the fact that my child continuously has to hear ‘Wow, you are really tall’, full of amazement: ‘You’re really, really, really tall!!!’ as if she is a criminal.”

Furthermore, unsupportive attitudes toward their physical appearance and inability to fully participate in school, sports, play and other leisure activities were addressed.

#### Parents’ perspectives on the impact of a child with MFS on parental life

##### Parental burden

Reported parental burden was attributed to multiple sources: high child care needs, lack of professional health care support, a limited social life and parental concerns about their child’s physical and psycho-social development.

First, high child care needs and care responsibilities were perceived as stressful. Parents felt highly responsible for their child’s health, were sometimes overprotective and feared not being present when something bad happened to their child.

A father (focus group 1, parent 5) reported, “My greatest fear is that if something goes wrong, I will not be there or I will not anticipate in the right way.”

Second, parents indicated unmet needs for parental support from the health care professionals involved in the care of their child with MFS. They experienced a lack of information about the current and future consequences of MFS on daily functioning of their child with MFS, as well as a lack of transparency in treatments and specialized care to improve their child’s daily functioning. Parents reported they felt unprepared for their parental tasks directly after their child was diagnosed with MFS. They indicated a need for tailored support in parental empowerment and access to extended information about the disease-related impact on daily functioning. Moreover, the parents desired professional support in how to discuss the diagnosis and future impact of MFS with their own children and the environment and in dealing with the mental health of siblings and themselves. Furthermore, the parents reported moderate support and understanding from friends and family; when given support, the parents valued it greatly.

Third, the adjusted and complex family schedules, parental duties and high care needs of the child with MFS all resulted in very limited parental leisure time. Consequently, the social life of the parents was limited, which added to the parental burden.

Fourth, future concerns about their child not being able to keep up due to physical deterioration and regular medical checkups were reported to be stressful. High-risk aortic surgery and early death were feared, especially when family members had already undergone this type of surgery. There were also concerns about standing out from peers because of their child’s physical appearance related to MFS, which provoked unsupportive attitudes from peers and adults. Furthermore, concerns about the impact of all this of on the child’s psycho-social development, behavior and coping were indicated.

A mother (focus group 2, parent 8) stated, “It is actually the emotional, psycho-social part that I am very worried about because she just cannot keep up with peers, and that really makes her sad, and it hurts to see that.”

##### Financial burden

Because of their children’s care needs, the parents regularly took days off from work, took unpaid parental leave, or were employed in a more flexible or less demanding and lower paying job than they were educated for. Additionally, travel expenses due to regular medical visits and higher expenses for clothing and shoes were mentioned.

#### Parents’ perspectives on the impact of a child with MFS on family life

##### Family burden

The high care needs of a child with MFS and additional care for other family members with MFS strongly affected the organization and health status of the entire family. Familial stress was induced by regular adjustments to complex family schedules. Therefore, family routines, leisure trips and holiday destinations were planned thoughtfully, and cancellations due to health problems experienced by their child with MFS led to great disappointment for all family members. Regarding this subject, one mother (focus group 2, parent 10) stated, “In the weekends when we have had a busy day, we need to rest the next day.

That is the same problem when we plan our holidays. When we leave for France, we have to drive a day and then rest for a couple of days, otherwise it’s not feasible for all of us.”

##### Family cohesiveness and caring

Strong family cohesiveness and caring about family members was perceived as a positive impact of MFS on family life. Family members supported each other with medical issues, concerns, and stressful events, and after complicated health problems, they divided tasks and bonded intensely.

##### Reproductive planning decision-making

Reproductive planning discussions between parents and within the family were complicated because of previous experiences. In the focus groups, the parents shared their experiences of their own pregnancies, discusses advances in assisted reproductive technology and discussed the consequences of MFS on the future life of a newborn baby with MFS and on future family life, as well as on the risk of pregnancy for a mother with MFS. Parents indicated their worries about having a newborn who has to live with the consequences of MFS and indicated the need for more extensive information in order to be able to make reproductive planning decisions. In addition, concerns about the future pregnancy choices of their children with MFS were addressed.

## Discussion

This study is the first on parents’ perspectives of the large impact of MFS on daily functioning of their child aged 4–12 years with MFS, themselves and their family.

“Cannot keep up with peers” was identified as an important theme in our study. Problems reported in previous studies of adolescents and adults with MFS regarding daily functioning were comparable to those in our parental reports. Adolescents and adults with MFS could not keep up with work, school and sports. Their participation restrictions were caused by pain, fatigue and physical limitations [3, 5, 9–15, 30]. These limitations had a negative impact on their physical and psycho-social development, as well as on their well-being in childhood [3, 5] and adulthood [9–13]. There are also comparable reports on children with various chronic and connective tissue diseases regarding restrictions in daily functioning, reduced quality of life and deterioration of physical performance, as decreased muscle strength, generalized joint hypermobility, increased pain levels, and decreased proprioception and stamina were all associated with decreased physical functioning and participation [16–20, 31].

“Being different” as a child with MFS and standing out from peers due to physical appearance related to MFS was also an important identified theme. In adults with MFS, unsupportive attitudes and insensitive teasing by peers due to their physical appearance were described and had consequences for their future social behavior in that they became more introverted and developed a lower self-esteem and self-image [32]. Likewise, a meta-analysis on children with various chronic diseases reported a less positive body image than their healthy peers [33], which might be a risk factor for lower self-esteem [34]. Another meta-analysis indicated that children and adolescents with visible signs of a disease and appearance-related features of the disease were more likely to be victims of bullying than their healthy peers [35]. Despite these factors, one study showed the quality of life in children and adolescents with MFS was not reduced; however, those children with more distinct physical MFS features (according to the systemic Ghent score [1]) had reduced emotional well-being subscales compared to children with a less distinct physical appearance [8]. A study on adults with MFS also reported a normal quality of life, although patients indicated that their lives would be significantly better without MFS, particularly in the areas of physical activity and self-image [12]. These findings indicate that ‘being different’ due to physical MFS features may affect psycho-social development, self-esteem and behavior and requires the attention of health care professionals involved in the care of children with MFS.

Parental burden was addressed by the parents in our study. The same holds for parents of children with chronic, congenital heart, and other connective tissue diseases who showed greater parental burden than parents and families of healthy children [36–41]. The sources were comparable: parenting stress and practical problems in daily life, high care child dependency, being chronically ill as a parent, having a limited support system, having a limited social life and having a minimum number of days on holiday. In our study, the parents reported an additional specific source of parental burden: concerns and fear of high-risk aortic surgery and early death, especially when family members had already undergone this type of surgery. The parental assessment of and attitudes towards the children’s functioning may be influenced by the parents’ fear of an adverse outcome will occur and stress regarding the diagnosis MFS. These concerns were also addressed by the parents of children with a congenital heart disease. Parents generally showed a higher incidence and severity of anger, anxiety, distress, depression, hopelessness and/or somatization symptoms than parents of healthy children [37]. Futhermore it should be noted that some of the parents are diagnosed with MFS themselves. It may be possible that these parents may extent problems they experience and/or have experienced in their own childhood to their child with MFS. This may cause overprotection and restriction of the child’s activities and participation. Therefore it is highly important to inform parents and children about MFS and discuss parental fears and attitudes towards MFS.

Parental burden also affects the family functioning. Families who had fewer psychosocial resources and lower levels of social support were at higher risk of psychological distress and lower family well-being over time [37, 38]. Furthermore, a meta-analysis showed that many dimensions of child well-being, such as problem behaviors, poor social competence and reduced pediatric quality of life, affected family functioning and burden as well [42]. Positive factors such as family cohesiveness and caring for each other reduced parental and family burden, as reported both by parents of children with a congenital heart disease [37, 38] and by our parents.

The parents indicated that family members had concerns about reproductive planning and decision making. These concerns have also been reported in parents with other hereditary connective tissue disorders, who indicated that the hereditary connective tissue disease significantly influenced reproductive decision-making because of the deterioration of personal health, increased consciousness of reproductive issues and chances in family life after having a child with MFS [43, 44].

### Study strengths, limitations and further research

The biggest strength of our study is that it is the first to describe the impact of MFS on daily functioning of children aged 4–12 years, their parents and family. The study was based on qualitative research in which trustworthiness and credibility as well as content saturation and verification were enhanced throughout the entire study period [22].

Our study has some intrinsic limitations. The focus group participants enrolled themselves without purposive selection for diversity of gender and parental diagnoses of MFS (as we did purposive selection for the interviews) [21]. To provide confidence that the identified themes captured all parental perspectives, member and expert checks took place thereafter.

Another limitation is that we used subjective parental observations of Dutch parents with a child with MFS. Questions about the generalizability of our findings to the perspectives of parents from other countries remain present although expert checks [22] with the members of the Marfan Europe Network endorsed all themes. Despite these limitations, we are confident that the qualitative method used and number of interviews and focus group participants were sufficient to identify themes concerning this unexplored research topic.

Our results indicate a need for professional awareness regarding the broad impact of MFS on daily functioning of children with MFS, parents and families. We are convinced that it is crucial for the health care professionals and multi-disciplinary teams involved in the care of children with MFS and their families to be aware of these parental perspectives so they can better understand and address the challenges and support needs of the entire family. Health care professionals should support and empower children to cope with and manage the impact of MFS on daily functioning and to optimize participation in school, sports, and other leisure activities. In this way, children with MFS will be better able to keep up with peers. Professionals should also take into account the impact of a child with MFS on parental and family life and discuss the possibilities of parental counseling and/or psychological interventions for all family members.

Our study provides direction for future quantitative studies on the impact of MFS on daily functioning of children with MFS, parents and families. The identified themes concerning the impact of MFS on daily functioning of children with MFS could form a framework to map the functioning of children with MFS in more detail and study the quantitative impact of these themes on a child’s daily functioning. A core set of objective physical pediatric measurements can be used to observe the clinical and functional outcomes in comparison with subjective reports of parents regarding daily functioning and burden of disease of their children. Additionally, surveys about pain, fatigue, functional performance, daily activities, participation, personal factors (behavior, self-esteem, and self-competence), environmental factors, burden of disease and quality of life in children with MFS are needed. Furthermore, surveys on parental burden and family functioning that investigate and quantify the impact of a child with MFS on parents and family are indicated. Finally, the parents reported the need for information about the consequences of MFS and the need for interventions to improve daily function of their children, themselves and their family. Hence, individual rehabilitation and educational programs may be tailored to empower and improve daily functioning of children and parents.

## Conclusion

Parents perceived a large impact of MFS on daily functioning of their children with MFS, themselves and their family. They reported that their children with MFS could not keep up with peers and experienced participation restrictions. Additionally, unsupportive attitudes towards their physical appearance related to MFS and inability to fully participate were addressed. Parental burden was caused by high child care needs, lack of professional health care support, a limited social life and concerns about development. Family burden was caused by adjusted and complex family schedules, other family members with MFS, and reproductive planning decision making. The parents perceived family cohesiveness and caring as positive factors.

More awareness among all professionals involved in the care of children with MFS and their families is needed so that professionals can address their support needs and provide tailored interventions, rehabilitation and/or educational programs to empower and improve daily functioning of the children, parents and family.

## Additional files

Additional file 1: COREQ (COnsolidated criteria for REporting Qualitative research) Checklist for “Marfan syndrome in childhood: Parents’ perspectives of the impact on daily functioning of children, parents and family.” (PDF 424 kb)

Additional file 2: Overview of key themes, subthemes/ICF-CY categories supported by quotes from parents: “Marfan syndrome in childhood: Parents’ perspectives of the impact on daily functioning of children, parents and family.” (PDF 94 kb)

## Data Availability

The data supporting the findings are contained within the manuscript and additional files. The anonymized datasets used and/or analyzed during the current study are available from the corresponding author on reasonable request.

## Abbreviations

ICF-CY: International Classification of Functioning, Disability and Health for Children and Youth
MFS: Marfan syndrome
