# Supplementary material for: Marfan syndrome in childhood: parents’ perspectives of the impact on daily functioning of children, parents and family; a qualitative study
Source: BMC Pediatr. 2019 Jul 29;19:262. doi: 10.1186/s12887-019-1612-6 (PMC6661807; doi:10.1186/s12887-019-1612-6)
Supplement: Supplementary file 1 — COREQ (COnsolidated criteria for REporting Qualitative research) Checklist for “Marfan syndrome in childhood: Parents’ perspectives of the impact on daily functioning of children, parents and family.” (PDF 424 kb) [file 12887_2019_1612_MOESM1_ESM.pdf]

Additional file 1
COREQ (COnsolidated criteria for REporting Qualitative research) Checklist for "Marfan syndrome in childhood: Parents' perspectives of the impact on daily functioning of children, parents and family."

This list is a checklist of items that should be included in reports of qualitative research. The page number of the manuscript "Marfan syndrome in childhood: Parents' perspectives of the impact on daily functioning of children, parents and family" is reported where each of the items, listed in this checklist, are considered.

Topic	Item No.	Guide Questions/Description	Reported on Page No.
**Domain 1: Research team and reflexivity**			
Personal characteristics			
Interviewer/facilitator	1	Which author/s conducted the interview or focus group?	2
Credentials	2	What were the researcher's credentials? E.g. PhD, MD	1; 2
Occupation	3	What was their occupation at the time of the study?	2
Gender	4	Was the researcher male or female?	2
Experience and training	5	What experience or training did the researcher have?	2
Relationship with participants			
Relationship established	6	Was a relationship established prior to study commencement?	2
Participant knowledge of the interviewer	7	What did the participants know about the researcher? e.g. personal goals, reasons for doing the research	2
Interviewer characteristics	8	What characteristics were reported about the inter viewer/facilitator? e.g. Bias, assumptions, reasons and interests in the research topic	2
**Domain 2: Study design**			
Theoretical framework			
Methodological orientation and Theory	9	What methodological orientation was stated to underpin the study? e.g. grounded theory, discourse analysis, ethnography, phenomenology, content analysis	2
Participant selection			
Sampling	10	How were participants selected? e.g. purposive, convenience, consecutive, snowball	2
Method of approach	11	How were participants approached? e.g. face-to-face, telephone, mail, email	2
Sample size	12	How many participants were in the study?	4
Non-participation	13	How many people refused to participate or dropped out? Reasons?	4
Setting			
Setting of data collection	14	Where was the data collected? e.g. home, clinic, workplace	4
Presence of non-participants	15	Was anyone else present besides the participants and researchers?	4

Description of sample	16	What are the important characteristics of the sample? e.g. demographic data, date	4; Table 2
Data collection			
Interview guide	17	Were questions, prompts, guides provided by the authors? Was it pilot tested?	2; Table 1
Repeat interviews	18	Were repeat inter views carried out? If yes, how many?	2; 4
Audio/visual recording	19	Did the research use audio or visual recording to collect the data?	2
Field notes	20	Were field notes made during and/or after the interview or focus group?	2
Duration	21	What was the duration of the inter views or focus group?	2
Data saturation	22	Was data saturation discussed?	2; 4
Transcripts returned	23	Were transcripts returned to participants for comment and/or correction?	4
**Domain 3: analysis and findings**			
Data analysis			
Number of data coders	24	How many data coders coded the data?	2
Description of the coding tree	25	Did authors provide a description of the coding tree?	2
Derivation of themes	26	Were themes identified in advance or derived from the data?	2
Software	27	What software, if applicable, was used to manage the data?	2
Participant checking	28	Did participants provide feedback on the findings?	4
Reporting			
Quotations presented	29	Were participant quotations presented to illustrate the themes/findings? Was each quotation identified? e.g. participant number	4,5,6; additional file 2
Data and findings consistent	30	Was there consistency between the data presented and the findings?	4
Clarity of major themes	31	Were major themes clearly presented in the findings?	4,5,6; figure 1; additional file 2
Clarity of minor themes	32	Is there a description of diverse cases or discussion of minor themes?	4,5,6,7,8; figure 1; additional file 2

This checklist was developed from: Tong A, Sainsbury P, Craig J. Consolidated criteria for reporting qualitative research (COREQ): a 32-item checklist for interviews and focus groups. *International Journal for Quality in Health Care*. 2007. Volume 19, Number 6: pp. 349 – 357
