# Supplementary material for: Marfan syndrome in childhood: parents’ perspectives of the impact on daily functioning of children, parents and family; a qualitative study
Source: BMC Pediatr. 2019 Jul 29;19:262. doi: 10.1186/s12887-019-1612-6 (PMC6661807; doi:10.1186/s12887-019-1612-6)
Supplement: Supplementary file 2 — Overview of key themes, subthemes/ICF-CY categories supported by quotes from parents: “Marfan syndrome in childhood: Parents’ perspectives of the impact on daily functioning of children, parents and family.” (PDF 94 kb) [file 12887_2019_1612_MOESM2_ESM.pdf]

Additional file 2: Overview of key themes, subthemes/ICF-CY categories supported by quotes from parents: “Marfan syndrome in childhood: Parents' perspectives of the impact on daily functioning of children, parents and family”

Key themes	Subthemes/ ICF-CY categories	Quotes (Respondent number)
Impact of MFS on daily functioning of children with MFS		
Cannot keep up with peers	school (d8)	▪ “The school days on which he has to attend school gym are actually the most difficult days for him. On Tuesdays, he starts off with school gym, then it's his lunchtime, and then, when his peers go outside to play, he can't keep up anymore and has to stay inside. School teachers noticed that a full school day is just not feasible for him.”(F2P10)
	sports (d9)	▪ “He does not like football at all because the other kids are just physically stronger and much faster, and then, he is always last to finish." (I2)
	leisure (d9)	▪ “He often looks a bit lost in the crowd, a bit..., a bit ashamed. For example, at a children's party he stands at a table, and they all want to grab something, then the other children are always first.” (F3P12)
	play with peers (d9)	▪ “When she comes home, she tells about her girlfriends who can run faster, and if they play tag, she will not manage to tap someone because she is not fast enough.” (F3P16)
	fatigue (b4)	▪ “We can undertake something together for half a day, but a full day is not feasible, and if we try a full busy day, then he gets overtired and has to throw up in the evening; that is really intense.” (F1P2)
	pain (b2)	▪ “She has 'the joint MFS', so her joints cause problems and pain. We are not able to go out for a full day, for example, if we go for a walk, after an hour she says: 'Mom, I suffer from my back and knees, and I am tired.' So, I experience that she is always in pain, and no, she does not complain but I see she suffers. And, then, I worry there is nothing to do about this pain, yes, pain medication, but I prefer not to give that to her because she is still so young. Then, professionals tell me, the heart is still not too bad….. but she is in pain all day.” (F3P15)
	mobility (d4)	▪ “She is a little less fast and a bit more clumsy than her friends. And, then, children in our neighborhood laugh at her when they are having cycling and running games together.” (F2P9)
	self-care (d5)	▪ “Tying his shoes takes a long time. We always have to wait for my son, so we are used to that now. At least he does it all by himself. And buttons..., that does not work well, for example, he has not enough strength to press buttons.” (I4)
Being different	personal factors	▪ “We noticed that she is starting to become a bit more insecure about certain things, such as her tall stature. She is now slowly getting aware of her body. She starts asking us questions from time to time, or becomes sad: 'I'm pretty tall, and how is that possible, and why do I not look like my girlfriends?' So, her physical awareness slowly starts, and that worries me.”(I7)
	unsupportive attitudes (e)	▪ “When she is on her knees, she needs 5 minutes to get up like an old woman, and then, there are children who say ugly things and laugh at her.” (F3P15)

Impact of a child with MFS on parental life		
Parental burden	concerns	▪ “I especially notice an increase in fatigue, and I worry about how much energy he needs, to be able to do his schoolwork. What will his energy level be at high school; can he really handle it, or should he take a step back? We will see.”(F2O10)
	lack of support	▪ “He is the first MFS patient in our family, and then, my family starts asking questions like: ‘Is he not too thin?’ and ‘Please give him food!’ I think children with MFS are already very thin and tall, and those remarks are not supportive for his self-confidence either, but how do I explain that to my parents and aunts?“ (I4)
	care needs	▪ “He needs a lot of care, and those therapies and appointments are always planned during school hours, never after. That is definitely not convenient, but it seems as if doctors always think that you, as a parent, just have all the time in the world.” (I3)
	limited social life	▪ “I think I really have to take into account my child’s energy level as well as the energy level of my partner with MFS, who also has limited energy. For example, today we're going to the hospital and tomorrow I will have to keep my children busy on my own because my partner has to rest; yes, I have a very busy life.”(I2)
Financial burden		▪ “Sometimes it is a lot of hassle, all arrangements. And, at work, my colleagues say: ’Do you really have to take a day off to go to the hospital with your child…again?’ I find that the hardest. Even though I arrange everything, I have to explain continuously. She has MFS, and no, I cannot arrange it any differently because those doctors only work on a certain day. Coincidentally, I also work on that day.” (I7)
Impact of a child with MFS on family life		
Family burden	family schedules	▪ “It affects our family activities because we are not able to do a lot of things because our son is tired quickly and cannot walk or cycle far distances.”(I9)
	other family members with MFS	▪ “I'm worried about the fact that there is a chance that he will not survive sooner or later, and that's what I'm worried about, that his sister by then is actually the only one left, and of course, I do not know what will happen to my own health. How long will I survive, and I'm worried that maybe in the future only mother and daughter remain, but you know when you are diagnosed with MFS, there's just a chance that something will go terribly wrong, especially with the heart.” (I1)
Family cohesiveness and caring		▪ “Our family slogan is always: ‘We know that we are special and that we are good at things and less good at other things, and that we have to take care of each other, and we do so.’(F2P7)
Reproductive planning and decision-making		▪ “I was done with pregnancies after we (my son and I) were diagnosed with MFS; I really do not want to pass on this disease to another child.” (I8)

ICF-CY, International Classification of Functioning, Disability and Health for Children and Youth. The ICF-CY uses an alphanumeric coding system. The letters used are “b” for Body Function, “s” for Body Structures, “d” for Activities/Participation and “e” for Environmental Factors and are followed by a numeric code that starts with the chapter number of one digit. I, interview; F, focus group; P, parent; MFS, Marfan syndrome.
